# Designing Robots for Elderly from the Perspective of Potential End-Users: A Sociological Approach

**DOI:** 10.3390/ijerph19063630

**Published:** 2022-03-18

**Authors:** Alina Betlej

**Affiliations:** Centre of Sociological Research on the Economy and the Internet, The Institute of Sociological Sciences, The John Paul II Catholic University of Lublin, 20-950 Lublin, Poland; alina.betlej@kul.pl

**Keywords:** age-friendly robots, robot design, needs of the elderly, ageing society

## Abstract

The proposed research aims to investigate the problem of age-friendly robot designing from the perspective of the potential end-users. The initial objectives addressed three main issues: how the elderly envision robots and their knowledge on technological development; age-friendly robot design; the elderly’s involvement in the robot design process. The empirical material analyzed are the results of in-depth interviews with people aged 70+. A sociological approach is proposed, based mainly on criticism of writing and the analytical and synthetic method. The theoretical framework is the perspective of an ageing society and technogerontology. The sociological approach enables better understanding of the sensitive problems of age-friendly robot designing from the individual point of view. It is concluded with a conceptual discussion on designing robots for the elderly. In particular, it is revealed how these issues could help in shaping social consensus about age-friendly technologies.

## 1. Introduction

Robotics is one of the fastest growing branches of the technology industry in recent times. The first assistive robots appeared in the 1950s to be enhanced by the Artificial Intelligence (AI) mechanism in the 1990s. Since then, researchers have focused on the potential of social robots [1,2]. Advanced robots are used now in many sectors, such as healthcare, military, education, social assistance, space exploration, etc. It is very difficult to identify an area of human activity in which such technological innovations are not used today. These solutions provide a rationale for future sustainability due to their specific potential for various applications in modern societies [1]. Humanoid robots, assistive robots or social robots regardless of how they are defined are currently in high demand [3]. These are seen as techno-social actors of digital transformation, with a particular focus on social relations and interactions in technology-driven ageing societies [4].

The issues of social relations and social interaction are central to this specific context of analysis [4,5,6]. Robots are designed to meet the needs of older people, supporting them in daily activities or enhancing their failing biological bodies [7]. In recent times, research on socially assisted robots has been very limited in some respects. A number of projects have looked at the development of robots for the elderly and identified specific functions that technological devices could perform in an ageing society [1,8]. It seems that technology itself is becoming not only an object of research, but also a personified subject of social change [4]. The possibilities of developing particular technological solutions, such as robots for the elderly, are continually discussed [7]. However, technological issues come to the forefront of analysis [7,9]. It is the human being who adapts to the requirements of new technologies. In this sense, technological rules today seem to be replacing social rules. The important ethical task is to reverse this perspective and postulate the necessity of adapting digital interfaces to real social needs. Therefore, the research was conducted to provide some insight to designers of advanced technological solutions for supporting elderly people in their daily activities. The aim of the study was also to give insight to the process of robot assessment by the elderly, to describe their needs of assistive robots, expectations of participation in the designing process as important end-users and to describe their visions for robots of the future in the context of an ageing society.

There is limited empirical qualitative studies devoted to these issues. In Poland, no research based on the in-depth individual interviews or focus groups has been conducted so far, which would address the problem of designing robots for the elderly. In the only quantitative study on humanoid acceptance, the authors used more general questions about people’s attitudes towards robots [1]. Poland is an example of an ageing society [6,10]. Older people are very often digitally excluded from using the modern technological tools intended for them due to low digital competences [6,10]. This problem became particularly evident during the COVID-19 pandemic, when older people had to make the digital leap. New technologies are being rapidly adopted by younger generations [6]. Analyzing future demographic forecasts, Poland will become a large market for such solutions [6,10]. Therefore, it is interesting to conduct the first qualitative study of this kind and find out the social, cultural and economic determinants of preferences for designing robots for the elderly, with references to the aforementioned theoretical frameworks related to human–machine interaction.

The paper is structured as follows: Section 2 outlines the theoretical background of robot development to support humans in the perspective of an ageing society, technology assessment and ethical issues. Section 3 provides an overview of the methodology, while Section 4 highlights the research findings. The discussion in Section 5 provides answers to the research question in the light of previous findings and theoretical backgrounds. Section 6 implies research limitations and directions of future studies. Finally, Section 7 brings the research conclusions.

## 2. Theoretical Background

The issue of the human–machine interface has been central to the interdisciplinary theoretical discussion since the early 20th century [4,5]. Especially, we have seen efforts to discover how this relation might have impacted the way of experiencing, perceiving, and subjectivity of humans [5,11,12]. The first thoughts focused on understanding the transformation of biological bodies under the influence of electronic machines [5]. The questions arose as to whether technology is a simple extension of biological bodies, or it transforms beyond the prosthesis to become a hybrid organism. The issue of discussions was also the ethical assessment of the extent that electronic technologies could be implicated in humans’ lives [13,14]. The peril of new technologies have come with the experience of being dependent on electronic tools and relying on machines in even simple activities [4,5,15,16]. The empirical works on people’s interactions with computers or other devices began to reveal the individual and social strategies for humanizing machines, establishing emotional relationships with them, trusting them and deploying to their home space [5,17,18]. The couplings between biological organisms and machines were also conceived as controversial because of the impact on human’s intimacy with a great power [8,13,14]. The early ideas of robotics did not emphasize machines as self-moving, self-autonomous, self-designing or self-achieving human’s goals [5]. 

We find the counter-narrative to this in arguments for posthumanism, which advocated our co-mingling with technology as a new techno-social entity [16,19]. The 21st century-robots have made the distinctions between human and machine thoroughly ambiguous [20,21,22]. Today, the boundaries between the natural and the artificial are blurring [4,23,24]. Modern robots are quintessentially microelectronic devices, invisible and hyperconnected with all spectrum of technological solutions used in contemporary societies [11,25]. Miniaturization has turned out to be about power, but we might confront this trend with the design of human-sized robots [7]. It is important to note that the effort to construct revolutionary robots to enhance humans have also met ethical doubts of scientists considering the ambivalent effects of technological development [13,14]. It was discussed how new devices could have impacted the issues of identity, gender, class, etc. [5,7]. Many authors have argued that humanity need reflexive strategies for the development of robotics [26,27]. They referred to social expectations of redefining boundaries between the world of humans and the world of technological devices.

It also could be acknowledged that no other perspective referring to technical categories is so epistemologically extensive. Considering changes with classes of subjectified processes (robotization, digitization, virtualization, hyperconnectivity) opens up spaces for the analysis of universal, typically human and humanistic issues. Technologies are social like a society being a technology [4,28,29,30]. The global sense of security for the future of the human condition in the world of technically mediated paradoxes has been disrupted [4,5,30]. The category of ties, communication or social relations has functioned in sociological thought for years [28]. It does not seem that in the next few years, man will change the way of entering society even more dramatically. On the other hand, human assistive technologies allow access to those techno-social areas and relationships which were previously beyond the limits of human cognition (emotions, spirituality) [12,31]. This kind of analysis which has appeared historically since the late of the 20th century enables us to notice some important attempts to answer the question about needs and expectations directed to the robot designers for elderly people. The questions of identity, boundaries and human–machine relations have influenced our debate on robots for older people [32,33,34]. We accept the thesis on ageing societies [6,16]. Advances in medicine and changes in lifestyle are leading to a longer life span. This has many ambivalent consequences. Older people require special individual and social care. On the other hand, we are observing rising social expectations of the elderly to improve their quality of life [6,10]. Technological development provides an interesting backdrop to such debates [9,24,35]. Thinking about robots for the elderly evokes many different associations. We can discuss many types of robots, assistive ones are only one example of solutions that can benefit older people [2,17]. We analyze a number of factors influencing the extent to which new technologies such as assistive robots are used [1,2].

The COVID-19 pandemic also brings a new perspective to research. There were very limited studies done in the socially assistive robots in the recent times [1,18,20]. This area still seems to be dominated by quantitative research, which limits the discovery of new social needs. Researchers specifically address only a few questions to robot designing processes [2,7]. They paid more attention to the social acceptance of new technologies among different age categories of end-users without rooting the discussion in the socio-cultural context [1]. The literature review on the ageing society directs attention primarily to three important aspects. The first is the digital divide and digital inequalities related to access to new technologies [21,36]. The second is the issue of digital competences and the ability of different countries to absorb, for example, assistive robots for the elderly [6,37]. The third is to assess cultural differences in the way such devices are perceived and used [38]. The results of technology assessment studies have repeatedly pointed to the inadequacy of technological innovations to meet the capabilities of their end-users, especially older people, who also live in developed societies [35,39,40]. In particular, researchers paid attention to the appearance issue and did not go far beyond the question [1,2,7]. The studies have been carried out in more developed countries where the digital index is higher [3]. They were focused on how robots could support the elderly in managing their daily activities, monitoring ambulatory assistance and in providing social interaction [2,8,12,17,23,24,34]. Social robots were mainly defined as materially embodied, autonomous actors that communicate and interact with humans on an emotional level [3]. They mimic typical social behaviors, states of mind, adapting to the demands of specific situations. The learning potential of such devices is currently under discussion [8,12]. Social robots can resemble humans, toys or animals [32,33]. The literature review highlights two predominant types of studies related to the issue of designing robots for older people. The first is associated with specific experiments and focus groups whose tasks were to discuss specific functionalities of robots [3,22]. The second is related to social acceptance in general and the factors determining the wider use of assistive or social robots in everyday life by older people [1,26]. It is commonly acknowledged, the widespread use of robots in elderly care is largely dependent on the social acceptance of new technologies [1,3,32,33]. Most of the studies were exploratory and it is difficult to draw general conclusions. However, a brief summary of the research findings gives some idea of the state of knowledge.

The previous studies have shown that assistive robots embodied and enforced the elderly, since these were not only simple extensions of biological bodies [1,3,27]. The use of robots has brought many benefits in the educational field and elderly care, especially in healthcare [27]. In many studies, older people emphasized the importance of independent living and improving teaching methods about new technologies for older people according to their competences [27]. They agreed that this would improve their quality of life and social relationships in many respects [2]. Moreover, in the previous studies, the authors explained how age-friendly robots were successfully used for various applications, such as companioning, assisting, playing, and overcoming users’ inabilities [26]. The research has also indicated that interactions between robots and the elderly could be improved for better outcomes [41]. Especially people’s acceptance of robots, motivation to use electronic devices and enjoyment in daily routines [3,20]. What is important, the previous findings suggested that highly smart robots seemed to be the least acceptable by older people, and elderly often raise many ethical considerations on age-friendly robots [3]. A separate field of studies is the literature on the ethical risks posed by the rapid development of new technologies for different categories of users [10,13,37]. The list of new potential social risks is growing exponentially [28,29,30]. The human–machine interaction has also brought many doubts for the elderly who preferred contacts with other people, instead of being dependent on robots [3,4,5].

The literature review context reveals interesting research gaps. There is still a lack of knowledge about the most important factors influencing older people’s attitudes toward age-friendly robots. Exploring criteria of robot aesthetics and functions could be a start point for discovering the role of the other mediating factors which are rooted in socio-cultural background. The second issue is the need to analyze the elderly expectations in participation in designing robots. This problem seems to be overlooked in the literature. The results of theoretical approach analysis bring us to the conclusion that there is also a cognitive gap relating to research on ethical considerations on age-friendly robots as interpreted by the elderly. The author aims to fill these knowledge gaps by providing a Polish elderly perspective on designing age-friendly robots.

## 3. Materials and Methods

### 3.1. The Design/Methodology/Approach

The primary data collection and research is based on a qualitative research method. It aims to provide rich descriptions of a selected phenomenon, such as how robots for the elderly are perceived, experienced, interpreted and evaluated by their end-users. The researcher assumes that the process of building a particular conception of social reality is closely related to the type of research conducted and the specificity of the analytical material collected (data are qualitative in nature). This approach identified as sociological enables the better understanding of the important social, economic and cultural issues of interest, such as adaptability, designing and implementation of new technologies in the context of an ageing society. The research analyses have been theoretically grounded, but the theory explains the different ways of interpreting human–machine interactions and helps identify possible contexts of analysis without structuring the research results. The approach is based mainly on criticism of writing and the analytical and synthetic method, i.e., a constructive approach which goes beyond the paradigm of cause-and-effect thinking and facilitates the introduction of idealistic ontological solutions, such as referring to the category of human imagination. The qualitative orientation of research seems to be the most appropriate for exploring the sensitive problems from the seniors’ point of view. The method provides more in-depth descriptions and analysis, such as the “how” and “why” [27]. The main premise of the research was to seek answers to the following questions (Supplementary Materials Table S1):

*How do they assess their digital competences? Which technologies do they use on a daily basis? How do they experience technology development? How do they imagine robots for the elderly? Do they feel the need to use such solutions? How do they perceive the opportunities and threats related to the development and dissemination of assistive robots? Why do they envision future technologies for seniors in such ways?*

The research focused on collecting detailed information on different spheres of respondents’ lives related to the use of new technologies. A grounded theory approach developed by [42] was used to investigate the sensitive issues of human–machine interaction in the specific people’s age context, which was interpreted rather in terms of a social and cultural phenomenon than only a simple demographic characteristic. The method links the theoretical explanations of the chosen phenomenon with the empirical research in order to understand it from the viewpoint of potential robot end-users. The data were collected through semi-structured in-depth, open-ended interviews (Supplementary Materials Table S1).

### 3.2. Participants/Sampling

In order to select the participants, the researcher followed the purposive sampling strategy of maximum variation [43], which was initiated by “identifying differential characteristics or criteria for constructing the sample” [43]. In this research, the identified varied characteristics were age, gender, place of residence and education level. The researcher recruited the participants using a snowball method, looking for people ranging in ages from 70 to 90, with different education levels and work experience, from a farmer to a university professor. A total of 10 participants took part in the study, including male (5) and female (5). The respondents came from different social backgrounds. What is important, all participants were healthy without cognitive impairment. They had problems typical of the elderly, such as poor eyesight and physical function. Participation was voluntary. There were no expectations for compensation. They were informed about the purpose of the study, the research data management procedure adopted by the researcher and about anonymity. The interviewees were also assured of their right to refuse to participate in or to withdraw from the study at any stage. They all have agreed to participate in the interviews. The participants are identified by codes in the study. The study protocol was approved by the Research Ethics Committee of the John Paul II Catholic University of Lublin (Table 1).

The second sampling method used in the study was theoretical sampling/data saturation [42]. The logic of this approach is based on the assumption that on the basis of the data that have been collected and analyzed hitherto, further data collection is unnecessary, as the implementation of additional interviews would not significantly contribute to solving the research problem. It is framed in grounded theory [43] and was defined by Glaser and Strauss [43] in these terms:

*The criterion for judging when to stop sampling the different groups pertinent to a category is the category’s theoretical saturation. Saturation means that no additional data are being found whereby the sociologist can develop properties of the category. As he sees similar instances over and over again, the researcher becomes empirically confident that a category is saturated. He goes out of his way to look for groups that stretch diversity of data as far as possible, just to make certain that saturation is based on the widest possible range of data on the category. According to these two sampling methods, the researcher continues to sample, collect data and analyse them until no new insights emerge from the ongoing sampling process.*

### 3.3. Data Collection

The current study is based on the semi-structured in-depth interviews (IDI) which were conducted in Poland in 2021 and early 2022 and ranged from 1.5 to 2 h. It was preceded by a pilot study of 4 people aged 70+ in October 2021 to validate the study protocol and research questions. The interviews were audio-recorded and transcribed verbatim. In one case, where the participant did not consent to be recorded, the interview was transcribed manually by the researcher during the interview. The transcription process took between 5 and 6 h for each interview. The transcripts were analyzed then. The purpose of interviews was to engage in dialogue with participants about their everyday experiences in using new technologies, knowledge about technological development, fears, needs and dreams of robots for the elderly. The interviews covered the same topics with the possibility of adding new problem issues important to the respondents or that emerged during the conversation. The researcher implemented elements of the biographical method, which allowed us to reach the subjective experiences, respondents’ life stories on using new technologies and crossing to digital revolution. The biographical lens of the narrators were important for understanding the phenomenon of robots for elderly people as perceived, interpreted and imagined through their individual perspective which draw the frames of a wide social context similar for all participants.

The accuracy of the transcription was checked again by the researcher at the end of the study. This procedure involved re-listening to the interviews and comparing the transcripts with the audio tapes of the interviews to correct any transcription errors. During the current analysis of the interviews with seniors, by the seventh interview about 90% of all codes were identified. This fact is explained in the literature as data saturation, i.e., a situation in which the implementation of additional interviews would not significantly contribute to solving the research problem [44,45]. It is therefore assumed that the sample size was not a limitation of the research objectives.

### 3.4. Data Analysis

The gathered data were very subjective and rich. Thus, the analysis entailed reading a large amount of transcripts looking for similarities or differences, finding themes and developing categories. The transcriptions of the interviews were read several times, following a narrative method that allows for reflexive understanding of the participants’ experiences. The collected data were evaluated using the chosen coding approach interpreted as conceptual abstraction involving the assignment of general concepts (codes) to individual incidents occurring in the data. Data analysis was framed within a qualitative grounded theory methodology as a dynamic, intuitive and creative process of inductive reasoning, thinking and theorizing. The study is based on three kinds of coding procedures: open, axial, and selective coding to analyze the data collected through the interviews [42]. Open coding was focused on the conceptualization and categorization of phenomena through an intensive analysis of the data. Axial coding was implemented to investigate the relationships between concepts and categories that have been developed in the open coding process. The last phase of selective coding was adopted in order to integrate the different categories that have been developed, elaborated, validated and mutually related during axial coding into one cohesive theory.

To reach this goal, the researcher focused on the exploration of values, meanings, beliefs, thoughts, experiences and feelings specific to the phenomenon under study. The transcripts were systematically searched and arranged to increase the understanding of how the participants perceive designing robots for older people. The process of data analyzing predominantly involved coding or categorizing the data and was the most important stage in the process. The researcher reduced the raw information, then identified meaningful patterns and hidden meanings from the data to build a logical chain of evidence. The data were subsequently assigned into categories of identified themes or topics compiled in the study. The process of conducting interviews and the analysis of their transcripts were carried out in Polish language.

## 4. Results

In total, the coding process yielded over 110 concepts, of which 90 relevant concepts were eventually used, creating a total of three categories. The number of concepts in each category ranged from 15 to 21. These categories include: (1) experience in using electronic devices; (2) robots functions and appearance; (3) designing elderly-friendly robots. The core category that emerged from these three categories is *elderly–robot interaction, education and wellbeing*. The categories that emerged are presented in the following section along with illustrative quotes from older people who took part in the interviews.

### 4.1. Experience in Using Electronic Devices

The first part of the interview was focused on digital competence issues. Surprisingly, most of the participants rated their skills as good enough but with raising a specific reservation. The respondents felt that their skills were sufficient for the digital devices they used every day.

*Nowadays, it’s hard for someone who doesn’t know how to use a smartphone or a computer to live. I’m not that good at it, but I’m still learning. I have to, because without it I couldn’t even register with a doctor. I have learned more in the last two years than I have in my whole life (laughs). COVID proved to me that everything can be learned.*

[PLM72]

Some only used simple smartphones, which also caused them problems due to the functionality not meeting their expectations.

*I can use a mobile phone. Not long ago I had trouble with it, but my grandson taught me how to dial numbers and read text messages. I tend not to text because I can’t see very well, but I can answer if I need to.* *These new models are not at all suitable for older people. Everything is so small and the glaring colours annoy me too.*

[PLF90]

A smaller number used a computer mainly for entertainment, such as computer games or social networking sites.

*[…] I have a lot of free time on my hands so I play computer games, read a lot, write on a computer or spend time on social networks such as Facebook.*

[PLM88]

Some were able to use video chat software. Those who had used computers to an advanced degree during their working life continued to use new devices to a much greater extent.

*I can’t imagine life without a computer. If my laptop broke today, I would rather buy a new one than spend money on something else.*

[PLF70]

Only a few were willing to learn new digital skills or opted in to benefit the learning programs for elderly and those were mainly women.

*I would not want to learn new digital skills. My point is, I know everything that I need.*

[PLM88]

*I would love to learn something new, for example how research can be done on the Internet, how to analyse big data.*

[PLF76]

*My grandchildren have had distance learning lessons and I have seen what it looks like. This is something I would definitely like to learn—how you can have these conversations where you can see the other person on camera and hear them. That is amazing. I would love to be able to do things like that myself. Now I have to use the help of other people. People become disabled because they don’t know how to use such devices by themselves. The more we know how to do everything ourselves, the longer we stay fully functional and independent. It gives you such a sense of security.*

[PLF71]

Many of them would have failed governmental programs on e-inclusion as they had no access to information published on websites. The more the individual digital competences were assessed, the more the elderly realized being left behind the access to new technologies because of its rapid development. In order to address the issue of digital inequalities in Poland, all the respondents agreed that the country lagged behind most of the European Union countries in terms of digital skills of not only the elderly, but also adults in general.

*Residents of more developed countries, such as Scandinavian countries, Germany have higher digital competences. This is influenced not only by the wealth of the society, but also by access to new technologies, learning computer science in primary schools, at university.*

[PLM80]

*In Poland older people have a low level of digital skills. Their education, professional experience, but not only, also their individual character, attitudes towards new technologies affect these.*

[PLM70]

During the interviews, they usually came to the conclusion that they are digitally excluded instead of what skills they had; these were not enough to keep up with the digital revolution.

*I think I know how to use a phone and a computer very well, but compared to younger people, I am far behind. I will never catch up with them, because technology develops too fast.*

[PLM81]

*Mobile phones, computers, robots, these are the attributes of youth and the future and good health.*

[PLF90]

Even people who only use simple smartphones with difficulties were aware about digital transformations. What is important, they felt to be digitally excluded and conscious of their inability to cross the digital gap. The social and cultural background is very important in that analysis. People who have crossed digital revolution aged 55–60 were more open to robots but it was strongly connected with their professional work. If someone used computers at work, he or she seemed to be more curious about new devices independently from age. The oldest participant was a farmer and was 90 years old. She had a very low level of digital competences, as declared, but had agreed these solutions would be popular in future. The respondents remembered the times of the beginnings of digital revolution in Poland when they had crossed the future without any preparation. These experiences resulted in thinking that there is no possibility to overcome the new digital divide looming.

*Every technology ages, more repeatedly than humans. Older people would have always been three steps behind.*

[PLF90]

### 4.2. Robots Functions and Appearance

During the second part of the interviews, the questions about robot functions, appearance, practical applications in daily activities allowed eliciting more discussion. The participants had common visions of robotics development. These came from pop culture, which captured sci-fi literature, films, television and clichés about technological societies. The topic became part of popular beliefs formed by the passing of available information through a thick filter of preconceived notions, prejudices, personal experiences and science. These common visions of crossing the future along with new technological development are an important part not only of common cultural experiences, but also learning and socialization, nowadays.

Living in the same cultural circle has impacted the way of thinking about new technologies.

*I have seen robots in films, on television. I have read about them in books, in newspapers. Although today I would even say that most robots appear in fairy tales and children’s games. Robots are not unusual. We are familiar with them, although we have never had direct contact with them. […] I say “we”, meaning older people in general, because I don’t know anyone who uses a real, big robot.*

[PLF70]

From the answers to the questions about future visions of robots, it might be implied that the participants have knowledge about development of new technologies. They are aware about the appearance of today’s robots and specific functions. They get information from TV, movies, literature, family and friends. It was interesting that they had knowledge about robots used by Japanese or residents of Scandinavian countries. These have no correlations with their level of digital competences. Even people who only use simple smartphones with difficulties, were aware about digital transformations. What is important, they felt to be digitally excluded and conscious of their inability to cross the digital gap. The social and cultural background is very important in that analysis.

The question on how do you imagine robots for older people, conjured up associations with humanoids and human-sized robots. The interviewees gave robots human traits in the descriptions which evoked strong emotions. The other solutions, such as a pet-robot or a cleaning robot, appeared in the background.

*When I imagine a robot, I see a large humanoid approaching me and saying something.*

[PLF76]

These devices were assumed as emotionally charged, but evoked rather emotions from fascination to ambivalence, but never fear or terror. They imagined robots as devices for helping, working for humans, pitching in with some housework, such as cleaning, writing, etc.

*New technologies are amazing!* *I saw a robot on TV that looked like a little baby, it was so cute.* *Anyway, it doesn’t matter, the most important thing is that they can help us with so many of the jobs that cause us problems. They can even save our lives.*

[PLF70]

It was further indicated that robots will be used to improve healthcare for the elderly. A robot was described strongly in the context of an ageing society. The world’s most boundary-respecting robot was seen as engaging humans’ brains to support cognitive mechanisms.

*That really has to do with our aging population (development of age-friendly robots).*

[PLM70]

The third context was providing the elderly acceptable quality of life, which meant not only housework and healthcare but also enjoying life within the atmosphere of security, dignity and feeling of other carers’ presence.

*I know that everyone talks about elderly care and immediately creates black scenarios. It is often forgotten that we, the elderly, also want to enjoy life and make the most of it. We have a lot of free time and would like to spend it in interesting ways. Why isn’t anyone asking about our leisure activities? If an elderly person enjoys relatively good health, and we know that this is a relative term, they would also like to live well. Not only in dignity, but also in well-being.*

[PLM72]

The participants underlined that solitude was the most difficult problem to overcome and here they saw the potential for a robot. None of them had used age-friendly robots before, with the exception of health apps on smartphones. They did not know anyone among their friends or family who had used such a robot.

*So far I accept a robot on the condition that it is not smarter than me.*

[PLM88]

The participants declared that they would accept a robot if it were offered to them. This would be possible on the condition that the robot would not be smarter than them and the human–machine boundary they knew was not crossed.

*I would gladly accept such a gift, because why not? I don’t understand why I wouldn’t accept a device that can help me. Younger people don’t understand this, but it comes with age when everyday activities start to make it more and more difficult for us. I am not talking about people who are very ill and who depend on the help of third parties for their daily lives. Do you think such a person would not accept a robot that would take care of them or help them in situations where they are embarrassed by another person, even a family member?*

[PLF76]

There were voices among the participants calling for the replacement of doctors, who often objectified the elderly, with robots that could be more precise in diagnosis.

*A robot-machine instead of a doctor would be a good solution. A medical machine on the model of an ATM machine. I put my hand inside. The machine is checking my vitals. Such a device would be more accurate and would make life easier for the elderly. Doctors treat old people like objects, so there would be no difference.*

[PLM88]

### 4.3. Designing Elderly-Friendly Robots

The participants discussed contemporary robots they had heard about or they had seen before as not adapted to the needs of older people in many respects. They claimed that robot designers should not include elderly in the process of new technologies development. In their opinions, the cost-effectiveness of robot designing would be achieved only through mass-scale production. That is the reason why designers did not include older people as end-users in the creation process.

*Production of robots for the elderly will only be viable on a mass scale, and their needs are very different. They depend on age, physical fitness and health status. It would be necessary to study the demand of small but specific groups of recipients, such as participants in universities of the third age, residents of retirement homes, beneficiaries of social welfare homes or participants in workshops organised by various foundations and associations as part of EU-funded projects.*

[PLM72]

However, despite the growing number of advanced robots, many people had a low level of digital skills and stayed behind the digital revolution. The devices were perceived as too complicated. The miniaturization trend was evaluated very negatively by the respondents.

*Now each successive model of phone or computer is getting smaller and smaller, and as I get older my eyesight, too. It’s not good.*

[PLF90]

The first suggestions when designing the robot’s software framework were to develop a more elderly-friendly solution, with functions in the form of simplified large buttons, allowing the robot to move easily in all directions and to give commands. The easier in usage would be a voice-activated or a motion-activated robot. The participants underlined that the touch responsive devices would be assessed more positively by the elderly.

*The design should be aesthetically pleasing, without flashy colours.*

[PLM81]

*I would prefer devices with large keys.*

[PLF90]

*Touch devices would probably be easier to use, but voice activated or motion activated are the future.*

[PLM88]

*Importantly, a robot should not have a typical robotic voice.*

[PLF76]

The respondents were convinced that modern robots make sounds similar to children’s toys which are annoying. A sentence was said, repeated several times, that robots must engage their brains.

*A robot should engage my brain.*

[PLM88]

*It should do something with my brain to stimulate it.*

[PLM70]

*It should teach my brain to function well.*

[PLF70]

Although respondents had never used advanced smart robots, they considered some technological functions to be obvious and thus not very innovative. Cleaning, healthcare were rated as very important, but the needs of older people also related to other aspects of quality of life, such as entertainment, leisure activities and maintaining cognitive well-being. Therefore, in future robots, they would look for new functions related to education, games (in accordance with a model of computer games) and exercises to support brain development. The respondents used the phrases:

*The robot should remind me of certain things.*

[PLF76]

*It should stimulate me to act, to be active, to do something. It is more important than cleaning functions.*

[PLF71]

The participants have also set the framework for something like a comprehensive policy on age-friendly robots in the European Union. As they felt, today robots are tools for wealthy people in more developed countries. In Poland, a few people could afford to buy a robot, as they thought.

They perceived the needs of the elderly in all of the European Union countries as similar.

*We differ in our digital skills and the level of income, but our needs are very similar because we are rooted in a similar culture.*

[PLF76]

They hoped that changes in gerontology politics in the EU would bring a solution for robot designers, so that elderly supported by robots would become more widespread. They expected robot designers to research the needs of small groups of old people to match their needs but with involvement of civil society organizations which provided support for the elderly in the frames of specific projects.

*Small consumer groups should be investigated. The demand for such devices is certainly high. If people knew the capabilities of such robots, if someone taught them how to use them, they would certainly live better every day.*

[PLM88]

So far, none of the projects in Poland has benefited the elderly in this context. Due to ethical considerations described in the literature, the participants were asked about the ethical issues in designing robots for the elderly. It should be stressed that they had no serious doubts and ethical considerations related to the topic of designing robots for the elderly.

*I am aware that many ethical issues are raised, but in my opinion, the end justifies the means*.

[PLM70]

*If robots help people, support them, benefit them, what kind of doubts can we have?*

[PLM80]

*I see more benefits. It is unethical to leave old people without any help. If robots do not harm human beings, there are no risks here.*

[PLF90]

The participants also declared they would accept electronic implants or other devices to enhance their biological bodies in ageing process and did not see any ethical problems in that case. They were open towards solutions which could help them live a good life without suffering from illnesses.

## 5. Discussion

Designing robots for the elderly is a complex process, related to many sociodemographic factors. The age category is not a definitive framework for analysis, but rather provides some direction for the study. The results suggest that age, health status, education, work experience, previous experiences of using electronic devices and sociocultural socialization towards new technologies play an important role in respondents’ individual attitudes towards robots and their expectations in its designing process participation. It is seen in the three identified categories: (1) experience in using electronic devices; (2) robot functions and appearance; (3) designing elderly-friendly robots. These results are in line with many previous studies on some points. However, many of the findings differ significantly from earlier findings on key issues.

The subject literature review gave many diverging options on that matter [9,23,46,47]. Importantly, sociodemographic factors have been shown to play an important role in the acceptance of robotic assistive technologies, as highlighted in many studies [11,24,31,34]. The elderly were often seen through the prism of negative stereotypes of old age and were not considered as an appreciated group of new technology customers [46]. The previous studies have asked questions about the acceptance of robots by older people in order to define the factors that influence the formation of certain attitudes [1,25]. The answers typically referred to sociodemographic factors. However, many designers and engineers do not take these differences into consideration, despite being aware of this weakness [32]. Sociodemographic factors are a very general term which contains many different areas of focus, especially in the case of designing robots for older people. As the previous research indicated, it is difficult to investigate these factors with small sample sizes [1].

This study rooted in a sociological viewpoint has proved that the specific research methodology enables us to show and to understand the importance of the way the sociodemographic factors could be considered by robot designers. The analysis showed that older people perceived differences in robot acceptance by health status rather than by age category, as in many cases, people’s needs are related to health. People who suffer similar diseases or have similar daily health problems would have similar needs of robot assistance instead of their age. Small sample size studies are most reasonable in order to focus on testing electronic devices prototypes to meet the needs of specific groups of older people. The study participants were open towards robots for older people. Furthermore, they declared that they would accept a humanoid robot to support their wellbeing. These findings significantly differ from the previous studies in Poland and in other countries. In the search for answers to such sensitive questions, the way of asking questions and the methodology are therefore very important. Survey questionnaires can be incomprehensible to older people, due to specific vocabulary and form of closed questions with the possibility of indicating only a few correct answers. In the case of older people, even the font size is an important factor [23].

The second explanation is rooted in the different sociocultural background [4,6,10]. Comparing these results only in an explanatory manner, it should be stressed that the findings framed the society’s development since ageing is a global phenomenon. The next generation of older people will differ significantly in their acceptance of new technologies and their expectations of assistive robots. The author has not found a study on older people’s visions of new technologies development, but it could be indicated that global socialization towards new technologies is an important part of learning about robots [9,23]. The interviewees mentioned shared visions of robot development drawn from pop culture. Even the participants with low levels of digital skills were familiar with visions of human–machine interaction. The interactions with robots could be interpreted through the prism of culture, e.g., socialization of global thoughts, visions, interpreting schemes, common knowledge assets. As many authors suggested, human–robot interaction is a context-dependent construct that could be influenced by the culture and the context in which the interaction occurs [1,24,31,34].

The results showed that people’s digital skills are an important factor encouraging or discouraging the elderly to use more developed electronic devices, but not in case of potential interactions with robots. Participants in the study considered the use of robots with simple and understandable functions, regardless of digital literacy scores. The previous research described older people as facing a higher risk of digital exclusion than the general population [6,10]. The research findings are somewhat similar, but the given conclusion draws attention to the role of education, socialization and human–robot interactions. The participants had knowledge about the development of new technologies. They were aware about the appearance of today’s robots. Furthermore, they seemed to be more familiar with various electronic devices than previous studies’ participants. Interestingly, they had knowledge about robots used by Japanese or Scandinavians, but these had no correlation with their level of digital competences.

Moreover, during the discussion, it became clear that methods often used in research were adopted in order to give knowledge on some general topics without going into details [1,34]. The approaches based on assessment of the specific robot, where research participants could see, or interact with such devices were more beneficial in practical implications. Even robots shown in pictures or on videos seemed to influence older people and encouraged more open attitudes [2,3]. The notion of interaction with robots seems to be becoming the most important factor in future studies on designing robots for the elderly. In the analyzed study, the open conversation on designing robots has also influenced the participants’ perception since the situations evoked while talking are not comparable to closed surveys. The findings are in line with the previous research that the attitudes towards a robot improved after people had interacted with it [46]. Although most of the studies have focused on a range of different psychological or economical characteristics as a crucial factor mediating the elderly’s interest in specific robots, it should be highlighted the role of evoking interaction with robots which has been already rooted in the cultural socialization towards the understanding of new technologies in terms of a global process which includes also media, literature, movies, etc. [2,3].

With regard to the methodological variety of earlier research efforts, the study showed that the results of a qualitative study gave different implications, especially when comparing with the Polish research [1]. This approach offered deeper insights into the feelings and perceptions of the elderly towards robots. The results revealed that the more experienced people are with electronic devices and the more they know about electronic solutions (from family, friends, movies, and literature), the higher might be their willingness to use them when needed. Education is of the similar importance as the experience. It is important to underline that studies on differences in new technology use between young and older age groups would not contribute significantly to the problem’s solution in that case. Stereotypes about ageing might influence the design process and contribute to developing the false views of elderly as potential robots end-users. The research findings differed significantly to the previous research in the elderly’s needs description, who were willing to use robots which would engage their brain to stimulate its development and entertainment [1,2,3]. The mediating factor in those needs’ expressions was the health status of the study participant as they all were in quite good health living independently in their homes without assistive help of the third parties. The other insights from the research are in line with the preliminary studies indicating that older people preferred simple functions of robots, except one condition [2,3,46,47]. The results of the literature and research review showed that robot’s appearance was a very important factor that influenced the acceptance of robots. The results drawn from the interviews analyses support different conclusions since the participants declared to accept a humanoid robot in their home settings. The ease of robot’s use was evaluated as the more important factor encouraging its acceptance than robot’s appearance. This study is explanatory in nature and is difficult to compare to quantitative oriented findings, but a gender factor could be analyzed here also differently. Women were less experienced with new technologies and with a lover level of digital competences than men, but familiarized with robots by the media, families were more open to learn new skills.

What is more, the findings referred to the previous conclusions that readiness of older people to take part in the robot’s designing process [2]. The similarities of statements are seen in the lack of indecision on how individual participation in cooperation with designers could look like. What is new, the participants saw the potential of nongovernmental organizations supporting older people in that process by organizing specific testing groups in people’s daily environments, e.g., at their homes or places of other activities, such as Universities of the Third Age in Poland, and mediating communication with robot designers. The differences might depend on the country’s policies. The more welfare state approach is accepted in the EU countries. The EU development strategies have influenced the members to implement new regulations in the field of gerontology. That was assessed by participants as the premise for projects dedicated to designing robots which would meet the specific elderly group’s needs.

The study has shown that it is vital to include end-users in the robot designing process because they can make important suggestions on how to avoid stereotyping or constructing inadequate older people’s needs. The assumption is that, in addition to sociodemographic variables, there are a plethora of moderating factors that remain unconsidered. Finally, and this is the most striking result of this study, the ethical considerations did not cause so much controversy as has been stressed in the literature review [5,14,15,19,25]. The potential end-users’ expectations to improve health and maintain good quality of life was a crucial frame for the willingness to accept assistive robots with respect of human rights and law regulations. The clue is to create conditions for elderly–robot interaction, education on electronic devices in the pursuit of older people’s well-being. This core category has emerged along with the analyses of the participants’ answers during the interviews and is named: the core category that emerged from these four categories is *elderly–robot interaction, education and wellbeing*. However, when analyzing the data, it becomes evident that the deployment of assistive robots for elderly is in an early stage in Poland and the findings show how important is the end-user involvement in the designing process with help of non-governmental organizations. This puts the focus on the individuals, their sociocultural background and education on new technologies.

## 6. Research Limitations and Directions for Future Research

The findings display some limitations due to the research method applied. The qualitative research does not provide data enabling a holistic and systemic approach to the problem. However, the chosen method seems to be the most appropriate for identifying a perspective of the elderly to know their needs on assistive robots. The most appropriate approaches to research are those associated with the humanistic stream, i.e., the ones in which emphasis is placed on understanding the way the elderly experience and perceive technological development. A micro-level approach seems to be the most appropriate in that context, despite the difficulty to make general comparisons. In the future, it is planned to conduct a focus group study among the elderly with mild mental disabilities.

Future research can continue to explore the impact of elderly–robot interaction on acceptance of age-friendly technologies. The findings indicate that participation in designing robots or testing specific solutions can lead to effective collaboration between end-users and providers of new solutions. In-depth interviews could be used as the basis for focus group research and experiments, where researchers might observe the process of elderly–robot interaction.

In-depth interviewing could be used by researchers to gain more insight into elderly’s perceptions and interpretations of learning new digital skills, and to show where the process can be improved. Besides, the findings suggest the need for developing appropriate surveys on acceptance of robots and other electronic devices by the elderly. Finally, providing the elderly as an important consumer group would let them voice their problems, needs, expectations and help them handle a digital environment, and adjust to their well-being.

## 7. Conclusions

In the context of designing robots for the elderly, ten interviews were done with the initial objective to address three main issues. The first was, how they envision human–machine interaction and what is their state of knowledge on technological development. The second, was the appearance and functions of the future robot. The third, concerns the involvement of the elderly in the robot design process as important future end-users. The interviews provide a number of conclusions, such as: the elderly had positive attitudes toward humanoid robots and electronic devices whose shapes resembled things they knew (humans, animals, smartphones, healthcare equipment). Furthermore, they preferred a voice-activated, a motion-activated and touch responsive robot with nice voices/sounds. In the future, the elderly would look for robots to engage their brain and stimulate its cognitive function to support their well-being, education, daily activity and leisure time (playing games). What is more, it stressed the need for micro-level research on elderly’s needs for age-friendly robots. Finally, the elderly opted for being involved in the robot design process. They saw the potential of robot designer cooperation with civil society organizations which support older people in many projects or other governmental agencies. What is important, the questions on ethical issues did not meet much interest of the elderly. The core category that emerged from the data analyses is *elderly–robot interaction, education and wellbeing*.

## Figures and Tables

**Table 1:** Socio-demographic characteristics of respondents.

Code *	Age	Gender	Education
PLF90	90	Female	Basic
PLF76	76	Female	Higher
PLF70	70	Female	Secondary
PLF75	75	Female	Higher
PLF71	71	Female	Secondary
PLM88	88	Male	Higher
PLM81	81	Male	Secondary
PLM70	70	Male	Secondary
PLM72	72	Male	Secondary
PLM80	80	Male	Higher

* Code = PL-nationality, F/M-gender, Number-age.

## Data Availability

The data supporting reported results can be found at the Institute of Sociological Sciences of The John Paul II Catholic University of Lublin. Availability: on researcher’s request accordingly to the politics of data management at The John Paul II Catholic University of Lublin: alina.betlej@kul.pl.

## Supplementary Materials

The following are available online at https://www.mdpi.com/article/10.3390/ijerph19063630/s1, Table S1: Individual in-depth interview scenario.
