# Supplementary material for: Designing Robots for Elderly from the Perspective of Potential End-Users: A Sociological Approach
Source: Int J Environ Res Public Health. 2022 Mar 18;19(6):3630. doi: 10.3390/ijerph19063630 (PMC8948980; doi:10.3390/ijerph19063630)
Supplement: Supplementary file 1 [file ijerph-19-03630-s001.zip › ijerph-1598897-supplementary.pdf]

Supplementary File

Table S1. Individual in-depth interview scenario

Designing Robots for Elderly from the Perspective of Potential End-users

**Topic**	**Specific issues (questions)**	**Observations**
**Digital skills**	**Develop the topic systematically**<br>- What digital devices do you use every day?<br>- For what purposes do you use them?<br>- How do you use them?<br>- What do you find difficult about using these devices?<br>- When did you start using these devices?<br>- Where did you learn to use them?<br>-Who helps you to acquire new digital skills?<br>-Do you remember when you first bought a smartphone, a computer?<br>-Would you like to learn something about using new technologies/using new devices that you have not used before?<br>-How would you rate the digital skills of older people in Poland?<br>-Why do you rate them this way?	
**Knowledge on technological development**	**Develop the topic systematically**<br>-Have you already heard about robots for the elderly?<br>-Do you remember where you first heard about such inventions?<br>-What was your education on new technologies?<br>-Did you learn about such devices at school, or rather from family, friends?	

	-Are you interested in the topic of technological development? What interests you the most?<br><br>-How do you see Poland from this point of view? Why do older people in Poland differ in terms of access to new technologies?	
**Robots for older people/ Visions of robots for elderly**	**Develop the topic systematically**<br><br>-When you hear the phrase 'robot for the elderly', what do you think of? What do you see? What does the robot look like? What does it do? Where is it placed?<br><br>- Do you think such robots actually exist and are used somewhere? Or is this a vision of the future?<br><br>- If someone offered you such a robot, would you accept it? Would you like to have such a device at home? What exactly should your robot look like and what would it be used for?	
**Robots appearance and functions**	**Develop the topic systematically**<br><br>-What should robots for older people look like? Why exactly as you describe?<br><br>-What features should robots for the elderly have? Using your chosen example, describe a robot that already exists (have you seen/heard of one) or one that you dream of (that you would like to have in the future)?<br><br>- Why did you choose such an example of a robot?	
**Designing age-friendly robots**	**Develop the topic systematically**<br><br>-How could the process of designing robots for older people be improved?<br><br>-Would you like to take part in such an experiment/project where you could actively participate in the design of robots for the elderly?	

	-What would your role be?	
**Robots and ethical issues**	**Develop the topic systematically**<br><br>-How do you perceive the opportunities related to the development and dissemination of assistive robots?<br><br>-How do you perceive the threats related to the development and dissemination of assistive robots?<br><br>-Would you see any ethical contraindications associated with using/designing/selling robots for the elderly?<br><br>- Why do robots for the elderly arouse so much emotion, especially in the context of ethical issues? Why might older people or their carers fear such devices?<br><br>-We find two types of statements in the literature, which one would you agree with:<br><br>1. Robots will in the future be available to rich people who can afford to buy them.<br><br>2. Robots will be available in the future to poor people who cannot afford to pay for human care	
